# First Paleogenetic Evidence of Probable Syphilis and Treponematoses Cases in the Brazilian Colonial Period

**DOI:** 10.1155/2018/8304129

**Published:** 2018-10-10

**Authors:** Lucélia Guedes, Ondemar Dias, Jandira Neto, Laura da Piedade Ribeiro da Silva, Sheila M. F. Mendonça de Souza, Alena Mayo Iñiguez

**Affiliations:** ^1^Laboratório de Biologia de Tripanosomatídeos (LABTRIP), Instituto Oswaldo Cruz, Fundação Oswaldo Cruz, Av. Brasil, 4365 – Manguinhos, Rio de Janeiro, RJ 21040-900, Brazil; ^2^Instituto de Arqueologia Brasileira, Estr. Cruz Vermelha, 45 – Vila Santa Teresa, Belford Roxo, Rio de Janeiro, RJ 26193-415, Brazil; ^3^Departamento de Endemias Samuel Pessoa, Escola Nacional de Saúde Pública Sérgio Arouca, Fundação Oswaldo Cruz, R. Leopoldo Bulhões, 1480 Bonsucesso, Rio de Janeiro, RJ 21041-210, Brazil

## Abstract

Despite interest in the origins of syphilis, paleopathological analysis has not provided answers, and paleogenetic diagnosis remains a challenge. Even venereal syphilis has low infectivity which means there are few circulating bacteria for most of the individual's life. Human remains recovered from the Nossa Senhora do Carmo Church (17th to 19th centuries) and the Praça XV Cemetery (18th to 19th centuries), Rio de Janeiro, Brazil, were subjected to* Treponema *paleogenetic analysis. Historical data point to endemic treponemal infections in the city, including venereal syphilis. Based on the physiopathology of* Treponema pallidum *infection, 25 samples, mostly from skull remains of young adults, with no visible paleopathological evidence of treponematoses, were analyzed. PCR with three molecular targets,* tpp*47,* pol*A, and* tpp*15, were applied. Ancient DNA* tpp*15 sequences were recovered from two young adults from each archaeological site and revealed the polymorphism that characterizes* T. p. *subsp.* pallidum *in a female up to 18 years old, suggesting a probable case of syphilis infection. The results indicated that the epidemiological context and the physiopathology of the disease should be considered in syphilis paleogenetic detection. The findings of* Treponema *sp. aDNA are consistent with historical documents that describe venereal syphilis and yaws as endemic diseases in Rio de Janeiro. Data on the epidemiological characteristics of the disease and its pathophysiology offer new perspectives in paleopathology.

## 1. Introduction

The geographic origin of syphilis, an infectious disease caused by the spirochete* Treponema pallidum* subsp.* pallidum* [1], is controversial. Three hypotheses have been proposed to explain the emergence of venereal syphilis. The pre-Columbian hypothesis proposes that syphilis and other human treponematoses originated in the Old World before New World contact but was misdiagnosed [2]. The Columbian hypothesis proposes that syphilis arose in the New World before the contact period and was transported to Europe by Columbus's sailors [3]. Lack of immunity against the disease facilitated rapid dispersion among Europeans. This hypothesis is supported by ethnographic reports of the use of medicinal plants for the treatment of the disease in the New World [4, 5]. Finally, the Unitarian hypothesis suggests that there is a single treponeme with wide global distribution, and that, due to differences in climate, geographic conditions, and cultural practices, it is expressed as different forms of the disease [6]. These include syphilis and the so-called nonvenereal or endemic treponematoses (ET): yaws (*T. pallidum* subsp.* pertenue*), bejel or endemic syphilis (*T. pallidum* subsp.* endemicum*), and pinta (*Treponema carateum*) [7]. All treponemes infecting humans exhibit biochemical, histological, microbiological, genetic, and antigenic similarities [4, 8]. Differences in incidence, geographical distribution, age at acquisition, main mode of transmission, clinical manifestations, capacity for invasion, and severity of late stage disease have been demonstrated [9–11]. Therefore, genetic analysis could be useful in the differential diagnosis of* Treponema* species and subspecies. Complete genomes of* T. p.* subsp.* pallidum* and* T. p* subsp.* pertenue*, which cannot be distinguished morphologically, show 99.8% identity [12]. Phylogenetic studies indicated that syphilis seems to have emerged in the Americas since* Treponema* spp. evolutionary rates are compatible with pre-Columbian times [13] and no evidence for European strains prior to the syphilis pandemic was detected [13, 14]. However, reports of pre-Columbian venereal syphilis outside the Americas and the possibility that ancient syphilis strains existed in the Old World [13, 14] imply that the topic of syphilis origin is unsolved.

Paleopathological differentiation of treponematoses is challenging. The osteological lesions caused by* Treponema* sp. and other infectious disease parasites are similar, and treponeme lesions are most pronounced in advanced stages [15, 16]. However, reproducible osseous patterns, including frequency in bone involvement, that support discrimination among treponematoses have been documented [17–19]. Ancient DNA (aDNA) analysis can be a useful tool for study of* T. pallidum* in archaeological samples and provides an opportunity to research the origins of syphilis. Kolman et al. [20] reported the detection of* T. p.* subsp.* pallidum* aDNA in a 200-year-old skeleton from Easter Island with a lesion typical of syphilis. This confirmed the first paleogenetic identification of venereal syphilis based on the polymorphism of the* tpp*15 lipoprotein gene that is specific to the subspecies. Montiel et al. [21] reported recovery of* T. p.* subsp.* pallidum* aDNA from bones of human neonates recovered from the crypt of La Ermita de la Soledad (16th and 17th centuries), Spain. The aDNA was detected by PCR using* tpp*15 and* arp* genes as molecular targets. Recently, Schuenemann et al. [22] used next-generation sequencing (NGS) for reconstructing genomes of* T. p.* subsp.* pallidum* and* T. p.* subsp.* pertenue* from skeletons recovered from Mexico City (17th and 19th centuries), which belonged to a perinate and infants with treponematoses manifestations.

Venereal syphilis is the most severe treponematoses, affecting various tissues and organs, including the nervous system and causing severe disability and death. It develops in three stages, and is characterized by long latent and low-infectious periods. The tertiary phase of the disease, when the typical gummatous and inflammatory bone lesions occur, is the least infectious phase. This might suggest that paleogenetic analysis could be fruitless in the study and diagnosis of treponematoses. The secondary but highly infectious phase of the disease could provide a diagnostic opportunity, although it cannot normally be identified in the osteoarchaeological record, since it is not associated with visible skeletal lesions [23, 24].

During endemic and epidemic periods, when a significant portion of the population is affected, the possibility of obtaining positive aDNA results for treponematoses by surveying skeletal keeps increases. In this study, human remains recovered from the Nossa Senhora do Carmo Church (17th to 19th centuries) and the Praça XV cemetery (18th and to 19th centuries), located in the city of Rio de Janeiro, Brazil, were submitted to* Treponema* aDNA analysis. Historical data point to endemic treponemal infections, including venereal syphilis, in the city. The investigation of bone samples from all available individuals provided the first paleoepidemiologic scenario from the Brazilian Colonial Period.

## 2. Materials and Methods

### 2.1. Ethics Statement

This study was submitted to, and approved by, the Research Ethics Committee Nacional School of Health Public (Escola Nacional de Saúde Pública-ENSP) [AMI1] (CEP number 12/2013). The bone materials are the property of the paleogenetic collection of the Laboratório de Biologia de Tripanosomatídeos (LABTRIP/IOC/Fiocruz) under the supervision of Dra. Alena Mayo Iñiguez, in collaboration with the Institute of Brazilian Archaeology (Instituto de Arqueologia Brasileira-IAB).

### 2.2. Nossa Senhora do Carmo Church Site

**Figure 1:**
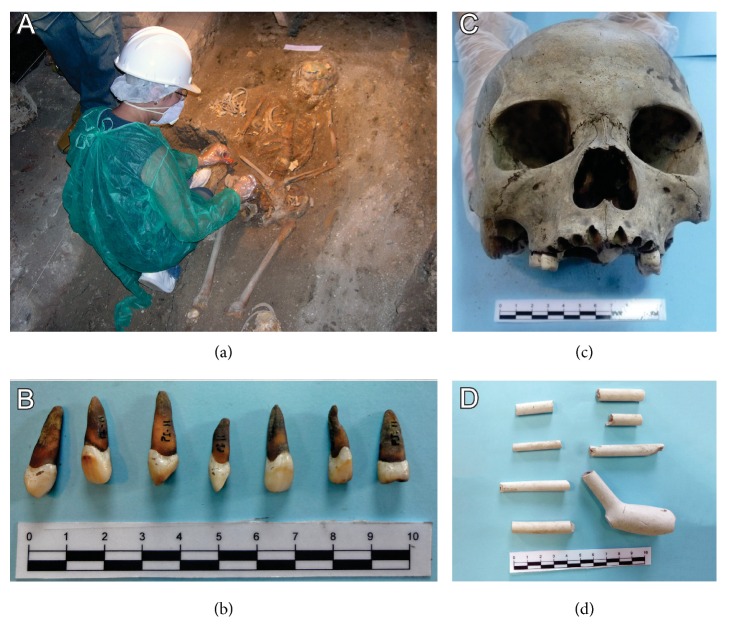
**Paleogenetic collection, human remains, and cultural artifacts found in the Rio de Janeiro archaeological sites. **(a) Procedure of paleogenetic collection of osteological samples in the INSC archaeological site performed by IAB archeologist, wearing individual protective equipment to preserve the integrity of archaeological remains; (b) a human skull from CPXV archaeological site; (c) teeth from CPXV individual, including intentional dental modifications; and (d) artifacts of African culture from CPXV archaeological site.

The Nossa Senhora do Carmo Church (INSC), or Old Cathedral, is located in Rio de Janeiro city, Brazil. The church became the royal chapel upon the arrival of the Portuguese royal family in 1808. In the same year, it was designated the cathedral of the city and remained so until 1976. In 2007, as part of the celebration of the bicentennial of the arrival of the Portuguese royal family, the chapel was restored [25]. During the work, several burials were discovered under the church floor, comprising Christian interments that took place until the mid-19 century [26]. The archaeological excavation was conducted by the IAB in collaboration with researchers of the Oswaldo Cruz Foundation (Fiocruz), Brazil. Articles relating to Catholicism (crucifixes) were found near or associated with the bodies buried in the nave area, and a few objects of African culture were found in the chapel area [25]. Bioanthropological analysis of skeletons* in situ *and paleogenetic collection of human remains were conducted by an anthropologist and a geneticist, respectively (Figure 1(a)). Bioanthropological analysis (n = 32) showed 13 of individuals were adults <35 years, 6 were mature adults >35 years, 5 were young <20 years, and 2 were children <10 years (IAB and PL/LABTRIP) [27]. The age was undetermined in the remaining 6. Eight individuals were identified as male and eight as female. Sex was undetermined in the remaining 16. The human matrilineal ancestry determined in 23/32 of the INSC individuals demonstrated that European ancestry was predominant (21/23) [27].

Paleogenetic collection involved recovering the archaeological remains in such a way as to avoid contamination during excavation as well as cross-contamination with modern DNA [28] (Figure 1(a)). It also prevented the degradation of aDNA from the time that remains were removed from their microenvironment at the archaeological site until the aDNA procedures were carried out at PL/LABTRIP [28, 29]. Measures taken during excavation included wearing protective clothing, gloves, head covering, masks, and use of sterile instruments [28, 30] (Figure 1(a)). Samples were collected in low intensity lighting and stored at 4°C protected from light in sterile containers. They were transported to the laboratory at 4°C and held at −20°C until paleogenetic analysis was performed [28].

Samples from burial, 31 comprising 14 skull fragments and 1 tooth (15 individuals), were selected for investigation. Bioanthropological data available from this study is in Table 1. Based on the physiopathology of* Treponema *infection, the criteria for sample selection were that they should be taken from young adults [31] and show no evidence of treponematoses, in the osteological material available. Lesions compatible with treponematoses considered in this study were extensive periostitis (thickened or spiculated), of symmetrical-type, mainly in the diaphysis of the lower limbs, especially in the tibia. We also searched for localized porosities in the skulls, associated or not with hypertrophic and confluent bone reactions, as* caries sicca *type, following [9, 17, 18]. These criteria were based on the following assumptions: (1) the clinical progress of the infection is such that the highly infective secondary phase of syphilis generally occurs in young adults, increasing the probability of detectable DNA, since the number of circulating spirochetes is high only in this phase of the disease [31] and (2) when the typical bone lesions are visible during the tertiary phase, the bacterial load is low. The lack of bone lesions increases the chance of DNA recovery [15]. Skull tissue was used, as another criterion of selection, based on ready availability and its tropism for* Treponema *sp. infection [32]. In the absence of the skull in one individual, a tooth was chosen as an alternate source of well-preserved genetic material [33]. Three subadults <18 years and 3 subadults <12 years buried on burial 31, which most were young adults (n=9), were also included in the analysis (Table 1).

### 2.3. Praça XV Cemetery Site

The Praça XV Cemetery (CPXV) was discovered and excavated, in 1996 during the construction of a tunnel at the port of Rio de Janeiro. The cemetery was known to have received bodies of the general population who died in major epidemics with an important African slave component [34].

Thirty-seven human skeletons were recovered from secondary burials dated from 18th century. During excavation, conducted by the IAB, complete skeletal series were not identified due to a high degree of anatomic disarticulation of individuals; instead, a series of types of bones (skulls (Figure 1(b)) and mandibles, for example) were collected [35]. After excavation, samples were kept at room temperature and protected from light and underwent a curation process of brushing the surface to remove soil without use of chemicals. According to bioanthropological analysis, (n=37) 22 of CPXV individuals were young adults <30 years, 10 mature adults >40 years, 4 adolescents <17 years, and 1 child <10 years (IAB and PL/LABTRIP). Seventeen were male and 14 female, and in 6 sex was undetermined [34]. Nine of twelve individuals from which tooth samples were available showed dental modifications (Figure 1(c)) consistent with African ethnic practices but also described in Amerindian groups [36]. Objects of African culture were found around the site, as well (Figure 1(d)) (IAB and PL/LABTRIP). African, European, and Amerindian haplogroups were verified in 25% of CPXV individuals [37]. For the present study, skull fragments, corresponding to 10 individuals, were obtained from the IAB, based on the selection criteria of the study and following the procedures described above. No evidence of treponematoses was observed in the osteological material available. Bioanthropological data available from this study is in Table 1. Eight individuals were young adults <30 years and 2 subadults <17 years.

### 2.4. Maintaining DNA Integrity

Rigorous precautions were taken to prevent aDNA degradation and contamination by modern DNA during collection and analysis at PL/LABTRIP where sample preparation, aDNA extraction, PCR, positive PCR controls, and post-PCR procedures including electrophoresis and sequencing were conducted at the Central Laboratory (LABTRIP/IOC/Fiocruz), which is located 500m from PL/LABTRIP [28]. The replication of experiments, including PCR, electrophoresis, and sequencing was temporally separated at the Central Laboratory (LABTRIP/IOC/Fiocruz). Extraction blank controls were processed in parallel with samples, and PCR negative controls were always included. The authenticity criteria consist of the absence of detectable PCR products (pPCR) in the sediment removed from area of the sacrum (archaeological site controls), extraction blank, and PCR negative controls. PCR positive controls were not present in the Paleogenetic Laboratory.

### 2.5. Ancient DNA Extraction

Prior to aDNA extraction, samples were decontaminated: the surface of the samples were removed and cleaned with 3% sodium hypochlorite. Subsequently, surfaces were irradiated with 254 nm ultraviolet light from a distance of 15 cm for 15 minutes [29]. After ultraviolet irradiation, bones were ground in an analytical mill using liquid nitrogen. Approximately 200 mg of bone powder was used for DNA extraction with proteinase K digestion as described by Iñiguez et al. [29]. Sediments (200mg) from the area of the sacrum of individuals from both INSC and CPXV, used as site environmental controls, were also submitted to aDNA extraction. For aDNA extraction, one blank control was included every six samples. The DNA IQ™ System (Promega) was used according to the manufacturer's bone extraction protocol with the modification of incubation at 56°C for 2 hours and the addition of 20 *µ*l of 0.2M EDTA and 10 *µ*l of proteinase K (20mg/ml) with gentle agitation. Alternatively, the QIAamp DNA Investigator-Qiagen kit was used according to manufacturer directions, with modifications: instead of the proteinase K solution specified by the kit, 30 *µ*l of proteinase K (Invitrogen) at 20 mg/ml was used; the incubation with TAE time was increased to 10 minutes at room temperature; and final centrifugation was at 17,000 x g for 2 minutes. The concentrations of aDNA were estimated at 260 nm absorbance on a Pico200 spectrophotometer (Picodrop™).

### 2.6. PCR and Sequencing

Three molecular targets were applied corresponding to the* tpp*47,* pol*A, and* tpp*15 genes of* T. pallidum *using primers and PCR conditions according to description of each genetic marker [20, 38, 39]. The targets allowed diagnosis of syphilis and ETs but did not discriminate among* Treponema *species and subspecies, with the exception of the* tpp*15 target, which discriminates venereal syphilis from ET [20]. PCR was performed in a total volume of 25 *µ*L, using Platinum* Taq *polymerase (Invitrogen, USA) in an Eppendorf Mastercycler® PCR Cycler (Eppendorf, Germany). The pPCRs were analyzed by electrophoresis in 1.5-2.0% low-melt agarose gels (Sigma, USA) and visualized under UV light, after staining with GelRed Nucleic Acid Gel Stain (Biotium, USA). All positive pPCR including those of unexpected length were submitted to nucleotide sequencing. The pPCRs were directly sequenced using BigDye Terminator v. 3.1 Cycle Sequencing Ready Reaction Kit (Applied Biosystems) in a 3100 Automated DNA Sequencer as recommended by the suppliers. Pairwise/Blast/NCBI and BioEdit v. 7.0.1 software were used for sequence analysis. Target sequences obtained were submitted to GenBank under accession numbers KU892169-70.

## 3. Results

**Figure 2:**
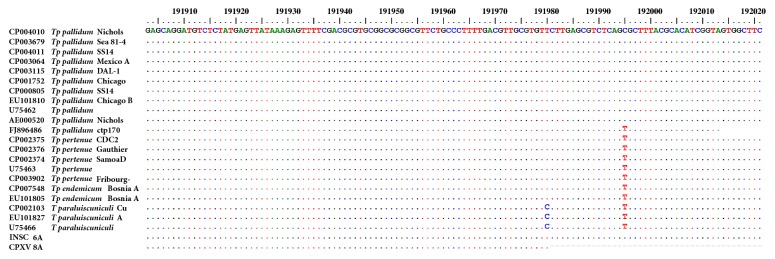
**Alignment of treponemal* tpp*15 target sequences**. Treponemal sequences recovered in this study compared to reference sequences available from GenBank.

One individual from INSC (ISNC6A) and one from CPXV (CPXV8A) were PCR positive for the* tpp*15 target. The remaining samples and controls, including aDNA extraction and PCR blanks and control sediments from the archaeological sites, were negative or presented nonspecific amplification. Nonspecific amplifications were sequenced, but no- or poor-quality sequences were obtained. Sequencing analysis of the individual from INSC when compared with reference sequence* T. p. pallidum *Nichols (Genbank CP004010) revealed the polymorphism T191995C that characterizes* T. p. *subsp.* pallidum *(Figure 2). The INSC individual with possible syphilis infection corresponded to a female up to 18 years old (Table 1). The individual from CPXV showed a short fragment in the* tpp*15 conserved region that confirmed* T. pallidum *and excluded* Treponema paraluiscuniculi *(T191980C) but did not allow* T. p. *subsp.* pallidum *discrimination. The CPXV individual probably affected by treponematoses was a female up to 17 years old (Table 1).

## 4. Discussion

Studies have shown the difficultly in recovering aDNA of* T. pallidum*. However, Kolman et al. [20] and Montiel et al. [21] were successful in using PCR to obtain genetic material of the bacterium using spermidine, a compound that stabilizes cell membranes and DNA structure [40]. Montiel et al. [21] reported that the primary factor in their successful* T. pallidum *aDNA detection was the focus on neonate remains with evidence of CS. According to the authors, the potential for aDNA detection is higher in young individuals, due to the greater number of spirochetes distributed throughout the skeleton. Schuenemann et al. [22] also attributed the potential successfully recovering bacteria aDNA when they focused the study on young individuals. These are the only studies that have been successful in PCR recovering* T. pallidum *genetic material. Bouwman and Brown [31] evaluated the presence of* T. p. *subsp.* pallidum *and* Mycobacterium tuberculosis *aDNA in 46 human bones, some showing evidence of treponematoses and tuberculosis. Analysis of bone samples collected from an English cemetery used from the 9th through the 19th centuries did not reveal* Treponema *sp. aDNA, while* M. tuberculosis *aDNA was obtained. The authors proposed differential aDNA preservation of the pathogens.* Mycobacterium tuberculosis *is the most commonly recovered pathogen in paleogenetic analysis, due to its high load in remodeled bone as well as the protection against DNA degradation provided by the thick and lipid rich bacterial cell membrane [31]. The lipid poor cell membrane in treponemes leaves the DNA vulnerable to degradation. Barnes and Thomas [41] analyzed* M. tuberculosis *and* T. pallidum *infections in human bones from museum collections dating from the 18th and 20th centuries. The authors concluded that an additional source of negative results in recovery of* T. pallidum *is its weak cell wall. Von Hunnius et al. [42] pointed to the lack of lipopolysaccharides, which are known to act as physical barriers to lysis, in the fragile outer cell membrane of* T. pallidum*. Spilgeman et al. [43] also discussed that the Gram-negative bacterium has high sensibility to temperature changes and easily lyses. In this study, we attributed the success of the aDNA* Treponema *detection to criteria adopted based on epidemiological data and mainly the pathophysiology of the disease.

Besides the age of remains, the type of soil, high temperatures, inadequacy on transportation, and stock conditions of sample after excavation are important factors that act in the aDNA preservation [43–45]. In this study, paleogenetic collection was applied on INSC excavation and samples were protected from the light and transported/preserved in low temperatures [27]. However, CPXV samples were obtained from IAB collection, where they were maintained at room temperature [34]. Both sites are localized in Rio de Janeiro with a tropical climate that interferes in the samples preservation [27, 34, 35, 46]. All these elements could explain negative results in most samples, but noninfected individuals cannot be ruled out.

The hypothesis of an American origin for both yaws and syphilis has been considered in many opportunities [13]. Pre-Columbian data of Brazil from Okumura et al. [47] and Eggers et al. [48] suggest treponematoses in coastal prehistoric groups dating from 2890 ± 55 to 2186 ± 60 BP and 5800-4500 BP, respectively. Filippini [49] observed bone paleopathological evidences suggestive of syphilis and yaws in Brazilian costal sites 2000-5000 BP. In many prehistoric North American sites, bone lesions confirming yaws and even suggesting venereal and congenital syphilis have been described [50–52] but urban and historical context in the present study must be considered.

In the present work,* Treponema *sp. aDNA sequences were recovered in two young females, and the polymorphism of* T. p. *subspecies* pallidum*, the causative agent of syphilis was observed in the INSC church individual. Positive molecular results are insufficient to confirm clinical and pathological conditions, but in the positive individuals, they strongly suggest active and infective treponematoses. Studies showed similar frequencies of disease in both groups of female and male, but varying according to age, with subadults, male, and adults as the most affected [18].

Yaws and syphilis were endemic in Rio de Janeiro during the colonial period. The former was especially associated with slaves coming from Africa [53]. Syphilis was particularly important in the colonial period and, along with yaws, was mentioned by Sigaud as a disease of Africans [53]. Yaws is expected to have affected many more people than syphilis, since infants and children were infected, while syphilis, acquired through sexual intercourse, affected a different age group. Children with CS rarely survived.

Herein paleogenetic results for treponematoses reported point to possible cases of both diseases, and the epidemiological and the historical context support the findings. Poor sanitary conditions contributed to the risk of treponematoses among African slaves, which predominated among the burials of the CPXV. One individual from CPXV revealed a* Treponema *sp. aDNA sequence that, based on historical and epidemiological records, could be consistent with either yaws or syphilis. As an endemic nonvenereal disease of tropical worldwide distribution, introduced in Brazil by African slaves, yaws is most probable, but syphilis should also be pondered. Since the CPXV archaeological site has a strong African component and African cultural evidence was verified in the INSC site, a high proportion of those individuals suffering from yaws, especially those of African ethnicity, is highly probable. However, it should be considered that there is evidence for all treponemal diseases in the South America since Pre-Columbian times [47, 54, 55].

According to Edler [56], yaws and syphilis were prevalent diseases among Europeans, Native Americans, and Africans in the Brazilian imperial period. The author states that health conditions of African people were deplorable and cites contemporary depictions that illustrate this scenario, including slaves suffering from yaws. Sigaud [53] reported that yaws, to which he refers as “piã,” was spread along the Rio de Janeiro coast in the 18th century, along with other deadly diseases. According to the author, these diseases were an inevitable consequence of the slave trade that established the exchange of lethal diseases between continents.

On the other hand, it is known that syphilis became epidemic during the Brazilian colonial period [57, 58]. Araújo [59] stated that high incidence of syphilis in Brazil, especially in Rio de Janeiro, has been reported by reliable sources since the 18th century. A survey by the government of Rio de Janeiro in 1798 revealed venereal disease as epidemic in the city [37]. In the 1860s, syphilis, tuberculosis, intestinal disorders, and intermittent fevers appeared in the annual reports of health officials of the empire as diseases disproportionally affecting the poorest. According to Araújo [59], high rates of mortality from syphilis have existed since the 18th century. In 1909, Souza [60] speculated that statistics on the incidence of syphilis could be dispensed with, since the rate of those affected was similar to the total population. Other authors have reported high mortality rates from syphilis during the Brazilian historical period [58, 61]. The possible syphilis-positive individual from the Nossa Senhora do Carmo Church site, an important church for the elite of the colonial/empire period, is consistent with historical data. Further investigational PCR assays to distinguish nonvenereal* Treponema *subspecies, using new diagnostic targets and/or methodologies [62] that could be applied in paleogenetic studies, are needed for a more comprehensive panorama of treponematoses in the Brazilian Colonial period. The paucity of molecular studies on* Treponema *spp. has been contrasted by NSG studies. Recent NGS data showed treponemal genomes recovery from asymptomatic bones and PCR negative samples [63]. In addition, NGS analysis of worldwide strains showed two genomic clusters, with the emergence of syphilis strains placed in the 18th century (mean calendar year 1733, 1588-1848) and the present-day epidemic clade, in the second half of 20th century [14]. The first application of NGS for treponematoses diagnosis in archaeological samples was recently demonstrated [22]. NGS technologies open up new possibilities on syphilis paleogenetic studies to better understand the paleoepidemiology of the disease.

This study reports the first aDNA detection of* Treponema pallidum *subsp*. pallidum *that indicates a possible case of syphilis in the Colonial Brazil. Distinct populations were included in the research. Since the INSC constituted mainly Europeans of the ecclesiastical class, and the CPXV included the general population, mainly Africans slaves, it seems that the vulnerability to treponemal infection was not limited to any social or cultural group. We suggest that systematic surveys of skeletal series considering paleoepidemiological modeling could help elucidate the origins and history of treponematoses, including syphilis, in the New World.

## 5. Conclusions

In this study,* Treponema *sp. aDNA sequences were recovered for the first time in individuals from the Brazilian Colonial period. The paleogenetic results that suggest* Treponema *sp. infection are consistent with historical documents describing venereal syphilis, as well as yaws, as endemic diseases in Rio de Janeiro. The findings in two young females indicated that not only the epidemiological context but also the physiopathology of the disease should be considered in syphilis paleogenetic studies.

## Figures and Tables

**Table 1:** Bioanthropological data of individuals of the present study.

**Sample**	**Age group**	**Sex**
INSC1a	Young adult	Female
INSC2a	Young adult	Female
INSC3a	Young adult	Female
INSC4a	12-15 years	Undetermined
INSC5a	30 years	Male
INSC6a	<18 years	Female
INSC7a	18-20 years	Female
INSC8f^*∗*^	<25 years	Female
INSC10a	<25 years	Female
INSC11	18-25 years	Female
INSC12a	10-12 years	Undetermined
INSC13a	Child	Undetermined
INSC16	18-20 years	Female
INSC17	<18 years	Undetermined
INSC18	10-12 years	Undetermined
CPXV6a	15-20 years	Undetermined
CPXV8a	<17 years	Female
CPXV9b	<15 years	Undetermined
CPXV10a	25-30 years	Male
CPXV12a	23-25 years	Female
CPXV15b	25 years	Male
CPXV16a	25-30 years	Male
CPXV17a	25 years	Male
CPXV20a	22-23 years	Male
CPXV28a	25-35 years	Male

^*∗*^A tooth was used in the absence of the skull tissue. Child: <12years; Young adult: <30 years following IAB protocol.

## Data Availability

The nucleotide sequence data used to support the findings of this study have been deposited in the GenBank repository (KU892169-70).
